# Parameter Selection in Coupled Dynamical Systems for Tomographic Image Reconstruction

**DOI:** 10.3390/jimaging12030126

**Published:** 2026-03-12

**Authors:** Ryosuke Kasai, Omar M. Abou Al-Ola, Tetsuya Yoshinaga

**Affiliations:** 1 Institute of Biomedical Sciences, Tokushima University, 3-18-15 Kuramoto, Tokushima 770-8509, Japan; yosinaga@medsci.tokushima-u.ac.jp; 2 Faculty of Science, Tanta University, El-Giesh St., Tanta 31527, Egypt; omr.aboualaela@science.tanta.edu.eg

**Keywords:** computed tomography, iterative reconstruction, parameter selection, coupled dynamical system, optimization

## Abstract

This study investigates the performance of image-reconstruction methods derived from coupled dynamical systems for solving linear inverse problems, focusing on how appropriate parameter selection enhances noise-suppression capability in tomographic image reconstruction. Our previous work has established the stability of linear and nonlinear variants of such systems on the basis of Lyapunov’s theorem. However, the influence of parameter choice on reconstruction quality has not been fully clarified. To address this issue, we introduce a parameter adjustment strategy based on an optimization principle. Two complementary optimization strategies are considered. The first employs ground-truth images to determine optimal parameter values that serve as a numerical benchmark for evaluating reconstruction performance. The second relies solely on measured projection data, enabling practical application without prior knowledge of the true image. Numerical experiments using phantoms with relatively high noise levels demonstrate that appropriate parameter selection markedly improves reconstruction accuracy and robustness. These results clarify how properly tuned reconstruction methods derived from coupled dynamical systems can effectively exploit their inherent dynamics to achieve noise suppression in tomographic inverse problems.

## 1. Introduction

Tomographic image reconstruction is one of the most representative examples of a linear inverse problem whose goal is to recover an unknown image from noisy projection measurements [1,2]. In clinical X-ray computed tomography (CT), accurate reconstruction is required under severe acquisition constraints such as low-dose or sparse-view scanning, where the influence of measurement noise becomes particularly pronounced [3,4,5,6]. Two major classes of reconstruction methods are commonly used: transform methods and iterative methods. Transform-based approaches, typified by the filtered back-projection (FBP) algorithm, remain the standard in many systems due to their simplicity and computational speed. However, when the CT inverse problem is severely inconsistent, transform methods are highly sensitive to noise and tend to produce degraded reconstructions. In contrast, iterative methods aim to reduce the discrepancy between forward-projected estimates and the measured projections through repeated updates of the image [7,8,9]. These algorithms are grounded in optimization principles and are capable of producing higher-quality images than transform methods, even when the inverse problem cannot be solved analytically. Representative examples include algebraic reconstruction techniques [10], the maximum-likelihood expectation–maximization (MLEM) [11] method, and multiplicative algebraic reconstruction techniques [12]. Recent advances in machine learning have also led to deep-learning-based reconstruction techniques. While such methods can offer improved image quality, their performance depends heavily on the training data and may lack robustness under unseen imaging conditions [13,14,15]. Consequently, iterative methods that provide robust, high-quality reconstructions without requiring large training datasets continue to play an essential role in practical CT imaging.

Recently, we proposed a class of coupled dynamical systems designed to simultaneously estimate unknown variables and suppress the influence of measurement noise in linear inverse problems [16]. The key idea is to introduce an auxiliary variable that models the measurement dynamics, thereby constructing a system of coupled ordinary differential equations in which both the estimate and a smoothed representation of the measured data evolve together. By discretizing a nonlinear variant of the continuous-time system by using a multiplicative Euler method, we derived a class of coupled reconstruction methods with a multiplicative structure, inheriting desirable properties such as stability and non-negativity. However, despite the established stability of the proposed systems, the influence of parameter selection on the quality of tomographic image reconstruction has not been fully clarified. To address this issue, we introduce a parameter adjustment strategy derived from an optimization principle. The goal of the present study is therefore to determine appropriate parameter values for the coupled reconstruction methods derived from the previously proposed dynamical systems so as to ensure well-behaved evolution and achieve high-quality reconstructed images.

To achieve this objective, we investigate two complementary optimization strategies. The first strategy employs ground-truth images to determine optimal parameter values that serve as numerical benchmarks for evaluating reconstruction performance. Tomographic reconstructions are performed using two numerical phantoms with different shapes. The resulting parameter values that minimize a ground-truth-based discrepancy are used as numerical benchmarks, thereby assessing whether the proposed optimization strategy can fully exploit the reconstruction capability of the coupled system. The second strategy relies solely on measured projection data, enabling parameter selection without prior knowledge of the true image and thus reflecting realistic clinical applications. Several evaluation functions are examined to verify the stability and reconstruction quality achievable by the coupled dynamical system under this practical setting. Furthermore, we introduce new evaluation functions specifically tailored to the coupled system that do not require ground-truth images.

Numerical experiments using phantoms with relatively high noise levels demonstrate that appropriate parameter selection markedly improves both reconstruction accuracy and robustness. These findings clarify how properly tuned coupled dynamical systems can effectively exploit their inherent dynamics to achieve strong noise-suppression performance in solving linear inverse problems.

## 2. Coupled Dynamical Systems for Tomographic Image Reconstruction

This section introduces the coupled dynamical systems that form the theoretical basis of the proposed reconstruction approach. First, we formulate the tomographic inverse problem and then briefly review the continuous-time coupled systems that motivate the proposed methods, followed by a detailed description of the discrete-time coupled systems used for reconstruction.

### 2.1. Problem Formulation

Tomographic image reconstruction can be formulated as a linear inverse problem, where pixel values $x\in R_{+}^{J}$ are estimated from projection data $p\in R_{+}^{I}$ according to the model,

(1) p=Ax+δ,

where $A\in R_{+}^{I\times J}$ denotes the projection operator and $\delta \in R_{+}^{I}$ represents measurement noise. Here, $R_{+}$ denotes the set of nonnegative real numbers. When the noise vector δ is identically zero and Equation (1) is satisfied by *x*, the inverse problem is said to be consistent in the sense of Hadamard [17]. In this case, the set

(2) $$E:=\left\{x\in R_{+}^{J}\;|\;p=Ax\right\}$$

is non-empty, and we denote an element of this set by *e*.

For later use, we define the normalization coefficients:

(3) $$\sigma_{j}=\sum_{i=1}^{I}A_{ij},\;j=1,2,\ldots ,J.$$

### 2.2. Continuous-Time Coupled Dynamical Systems

To address the inverse problem by formulating it as a coupled dynamical system, we consider continuous-time systems in which the image estimate and an auxiliary variable evolve simultaneously. Let $x\left(s\right)\in R_{+}^{J}$ denote the image estimate evolving with respect to continuous time *s*, and let $y\left(s\right)\in R_{+}^{I}$ represent an auxiliary projection variable that models the measurement dynamics [16]. The key idea is to introduce this auxiliary variable so that the evolution of the image estimate is coupled with a smoothed representation of the measured projection data. This coupling leads to a system of ordinary differential equations in which both variables influence each other over continuous time. Linear and nonlinear variants of such coupled dynamical systems have been proposed in our previous work, and their convergence properties were analyzed using Lyapunov-based arguments. In particular, appropriate Lyapunov functions were constructed to show that the coupled systems exhibit well-controlled dynamical behavior around their equilibrium points, ensuring robustness with respect to measurement noise. These continuous-time models provide the conceptual foundation for the discrete reconstruction methods introduced below.

### 2.3. Discrete-Time Coupled Reconstruction Methods

Here, we derive discrete-time reconstruction algorithms from the continuous-time coupled dynamical systems introduced in the previous subsection. The continuous-time formulation provides a *coupled dynamical system*, whose time discretization yields a *coupled dynamical method*. By applying an explicit Euler discretization with a fixed time step of one, this method is realized as explicit iterative update rules for both the image estimate and the auxiliary projection variable, resulting in discrete-time *coupled reconstruction algorithms*.

Let n=0,1,2,…,N−1 denote the discrete iteration index. The continuous-time variables x(s) and y(s) are represented in discrete time by $z\left(n\right)\in R_{+}^{J}$ and $w\left(n\right)\in R_{+}^{I}$, respectively. Here, z(n) corresponds to the reconstructed image at iteration *n*, while w(n) represents an auxiliary projection that evolves jointly with the image estimate. The initial values are chosen such that $z_{j}\left(0\right)>0$ for all *j* and $w_{i}\left(0\right)=p_{i}$ for all *i*. All update rules are applied component-wise for j=1,2,…,J and i=1,2,…,I. The resulting discrete-time algorithms preserve the coupling structure of the original continuous-time models and form the coupled reconstruction algorithms examined in this study:

(1) Linear Discrete Reconstruction Algorithm.

The linear reconstruction algorithm is obtained by applying the additive Euler discretization to the linear coupled ordinary differential equations. The resulting update rules are given by

(4) $$\begin{array}{ccc} z_{j}\left(n+1\right) & = & {\left(z_{j}\left(n\right)+\sigma_{j}^{-1}\sum_{i=1}^{I}A_{ij}\left(w_{i}\left(n\right)-A_{i}z\left(n\right)\right)\right)}_{+},\\ w_{i}\left(n+1\right) & = & {\left(w_{i}\left(n\right)+\tau \left(A_{i}z\left(n\right)-w_{i}\left(n\right)\right)+\nu \left(p_{i}-w_{i}\left(n\right)\right)\right)}_{+}, \end{array}$$

where ${\left(\cdot \right)}_{+}:=\max\left\{\cdot ,0\right\}$ denotes the projection onto the nonnegative orthant.

(2) EM-Type Nonlinear Discrete Reconstruction Algorithm.

The second algorithm incorporates multiplicative updates inspired by the expectation–maximization principle. The corresponding iteration is given by

(5) $$\begin{array}{ccc} z_{j}\left(n+1\right) & = & z_{j}\left(n\right)\;\sigma_{j}^{-1}\sum_{i=1}^{I}\frac{A_{ij}w_{i}\left(n\right)}{A_{i}z\left(n\right)},\\ w_{i}\left(n+1\right) & = & w_{i}\left(n\right){\left(\frac{A_{i}z\left(n\right)}{w_{i}\left(n\right)}\right)}^{\tau }{\left(\frac{p_{i}}{w_{i}\left(n\right)}\right)}^{\nu }. \end{array}$$

(3) MA-Type Nonlinear Discrete Reconstruction Algorithm.

The third algorithm employs exponential updates and is motivated by multiplicative algebraic reconstruction techniques. The update rules are

(6) $$\begin{array}{ccc} z_{j}\left(n+1\right) & = & z_{j}\left(n\right)\exp\;\left(\sigma_{j}^{-1}\sum_{i=1}^{I}A_{ij}\log\;\left(\frac{w_{i}\left(n\right)}{A_{i}z\left(n\right)}\right)\right),\\ w_{i}\left(n+1\right) & = & w_{i}\left(n\right)\exp\;\left(\tau \left(A_{i}z\left(n\right)-w_{i}\left(n\right)\right)+\nu \left(p_{i}-w_{i}\left(n\right)\right)\right). \end{array}$$

In all three algorithms, the parameters τ and ν are tunable and play a crucial role in regulating the interaction between the image update dynamics and the auxiliary-variable dynamics. Moreover, if τ=0 and the initial value is chosen as $w_{i}\left(0\right)=p_{i}$, then $w_{i}\left(n+1\right)=p_{i}$ holds for all n=0,1,2,…,N−1. Consequently, the coupled system reduces to an iteration involving only the image variable z(n), where the projection-domain variable is fixed to the measured data. In this case, each algorithm reduces to its corresponding standard reconstruction iteration.

For the EM-type and MA-type algorithms, the iterations evolve in an invariant set characterized by $z_{j}\left(n\right)>0$ and $w_{i}\left(n\right)\geq 0$. If the image variables are initialized with strictly positive values, the update structure preserves strict positivity of $z_{j}\left(n\right)$ for all *n*, so that the denominators appearing in the update formulas remain positive throughout the iterations. The auxiliary variables are initialized as $w_{i}\left(0\right)=p_{i}$ with $p_{i}\geq 0$. If $p_{i}=0$, then the update rules imply $w_{i}\left(n\right)=0$ for all *n*, so that the zero components form an invariant set of the dynamics.

## 3. Parameter Selection via Optimization

In this section, we formulate a parameter selection strategy for the coupled reconstruction methods based on an optimization principle. The objective is to identify parameter values that yield effective and well-behaved reconstruction performance by using evaluation functions that can be defined with or without access to ground-truth images.

### 3.1. Problem Setting for Parameter Selection

The proposed method focuses on iterative reconstruction algorithms with adjustable parameters and provides a way to explore parameter values that yield the most effective performance for a set of projection data sharing common characteristics, such as similar noise levels and internal structures [18].

First, given *K* projections $Y\in R_{+}^{I\times K}$ with the same number of elements *I*, we define an iterative algorithm with the target system *f* to determine the corresponding composite state variables $\Psi \in R_{+}^{(J+I)\times K}$, consisting of the image variables and the auxiliary projection variables, as follows:

(7) $$\begin{array}{ccc} & & \Psi_{k}\left(n+1\right)=f\left(\Psi_{k}\left(n\right);\lambda \right) \end{array}$$

for n=0,1,2,…,N−1 and k=1,2,…,K. Here, $\lambda \in R^{M}$ represents the parameters to be adjusted. For example, when the target *f* is the discrete systems used in this paper, M=2 and

(8) $$\lambda =\left(\begin{array}{c} \tau \\ \nu \end{array}\right).$$

Next, starting from the initial value $\Psi_{k}\left(0\right)$ (k=1,2,…,K) according to Equation (7), after *n* iterations (n=1,2,…,N), we define

(9) $$\Psi_{k}\left(n\right)=:f^{n}\left(\Psi_{k}\left(0\right);\lambda \right)$$

for k=1,2,…,K. We then seek the parameters that minimize the evaluation function,

(10) $$g\left(\lambda ;N\right):=\frac{1}{K}\sum_{k=1}^{K}d\left(f^{N}\left(\Psi_{k}\left(0\right);\lambda \right)\right)$$

where *d* is the distance function, defined below, evaluated at the *N*th iteration. Our goal is to find the elements of the set,

(11) $$\underset{\lambda }{\mathrm{argmin}}g\left(\lambda ;N\right).$$

Note that the function *g* depends on λ through the computation process of Ψ(N), which is obtained by iterating f(·;λ), where *f* includes λ as a parameter.

### 3.2. Evaluation Functions and Distance Measures

The experiments conducted in this study examined several types of distance functions, depending on the available information and the purpose of evaluation. These functions can be classified into three categories: image-based discrepancies that require ground-truth images, projection-based discrepancies that use only measured data and the forward operator, and coupled discrepancies that additionally incorporate the auxiliary projections appearing in the coupled dynamical systems. For each dataset *k*, the state variable is denoted by $\Psi_{k}=\left(x_{k},y_{k}\right)$, where $x_{k}\in R_{+}^{J}$ represents the image component and $y_{k}\in R_{+}^{I}$ represents the auxiliary projection variable.

First, when the true image e∈E is available, we use the Euclidean distance in the image domain:

(12) $$d\left(\Psi_{k}\right):=\|e-x_{k}\|,$$

which provides an ideal criterion for directly assessing the reconstruction accuracy of the image component. This ground-truth-based discrepancy is used as a numerical benchmark to evaluate the intrinsic performance of the reconstruction algorithms under controlled conditions.

Next, to measure the discrepancy between the forward projection and the measured projection $Y_{k}$ (k=1,2,…,K) without using the true image, we consider the following projection-based distances:

1. Euclidean distance:
  (13) $$d\left(\Psi_{k}\right):=\|Y_{k}-Ax_{k}\|,$$
  which we denote by Y2N.
2. Kullback–Leibler (KL) divergence:
  (14) $$d\left(\Psi_{k}\right):=\mathrm{KL}\left(Y_{k},Ax_{k}\right),$$
  which we denote by YKL, where KL(α,β) denotes the generalized KL-divergence [19] between nonnegative vectors α and β, defined by
  (15) $$\mathrm{KL}\left(\alpha ,\beta \right):=\sum_{i}\left(\alpha_{i}\log\frac{\alpha_{i}}{\beta_{i}}+\beta_{i}-\alpha_{i}\right).$$
  We also extend the definition by setting KL(α,0)=+∞ and KL(0,β)=β.

These criteria quantify the data fidelity between the measured projections and the forward projections of the image component in the current state, but do not explicitly reflect the internal structure of the coupled dynamical systems.

Finally, motivated by the Lyapunov functions introduced for the continuous-time coupled dynamical systems, we introduce coupled discrepancies that involve both the forward projection $Ax_{k}$ and the auxiliary projection $y_{k}$. For the squared Euclidean case, we define C2N as

(16) $$d\left(\Psi_{k}\right):=\tau^{0}\;{\left\|Ax_{k}-y_{k}\right\|}^{2}\;+\;\nu^{0}\;{\left\|Ax_{k}-Y_{k}\right\|}^{2},$$

and, for the KL-divergence case, we define CKL as

(17) $$d\left(\Psi_{k}\right):=\tau^{0}\;\mathrm{KL}\left(Ax_{k},y_{k}\right)\;+\;\nu^{0}\;\mathrm{KL}\left(Ax_{k},Y_{k}\right).$$

Here, the positive weights $\tau^{0}$ and $\nu^{0}$ are fixed parameters that control the balance between the fidelity to the measured data and consistency with the auxiliary projections. In the numerical experiments, we set $\left(\tau^{0},\nu^{0}\right)=\left(0.9,0.1\right)$.

Equation (12) is used only when the true image is available and serves as a reference measure of reconstruction quality. In contrast, Equations (13) and (14) provide projection-based baseline criteria that can be evaluated without ground-truth information. The coupled discrepancies in Equations (16) and (17) can also be evaluated from projection data and the auxiliary projections and are designed as one-variable surrogate functions derived from the Lyapunov functions of the continuous-time systems, thereby preserving the stability structure of the original dynamical formulation. In the parameter optimization associated with the optimization problem in Equation (11), the initial values of the tunable parameters are set to τ(0)=0.5 and ν(0)=0.5.

## 4. Numerical Experiments

In this section, we investigate how appropriate parameter selection enhances the noise-suppression capability of reconstruction methods derived from coupled dynamical systems for tomographic image reconstruction. The reconstruction method under consideration is an EM-type nonlinear discrete system (CDEM) (5). First, we examined the necessity of proper parameter tuning and its effect on noise suppression using numerical phantoms, where ground-truth images are available for quantitative evaluation. Next, we determined optimal parameter values based on several choices of evaluation function and identified which evaluation criteria are best suited for achieving effective noise suppression in tomographic reconstruction. Finally, using a physical phantom scanned by a clinical X-ray CT system, we assessed whether the proposed parameter selection strategy remains practically applicable when only measured projection data are available and no prior knowledge of the true image is provided.

### 4.1. Experimental Method

**Figure 1:**
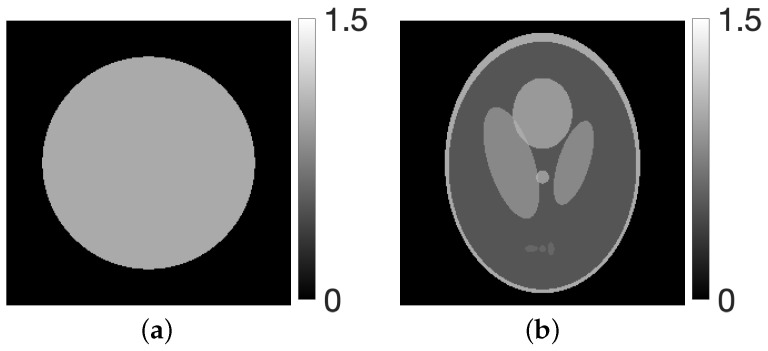
(**a**) Disc and (**b**) modified Shepp–Logan phantom images.

To investigate the effect of parameter selection for λ=(τ,ν) in the CDEM reconstruction method, numerical reconstruction experiments were conducted using ground-truth images. As shown in Figure 1, a circular disc phantom as well as a modified Shepp–Logan phantom were employed as the true images $e\in R_{+}^{J}$. The experimental setup simulates a CT scan with a 180-degree rotation, in which an image of size J=256×256 is reconstructed from projection data consisting of I=184×90 rays corresponding to 184 detector bins and 90 projection angles, while the supplementary sensitivity analysis includes an additional experiment with 45 projection angles to evaluate the influence of sparser sampling. To evaluate the influence of the tunable parameters, parameter planes were generated using projection data contaminated with additive noise whose variance was adjusted to yield a signal-to-noise ratio (SNR) of 20 dB for the main experiments, and 10 dB for the supplementary sensitivity analysis. These parameter planes enable a visual assessment of regions in the (τ,ν) space that yield effective reconstruction performance at a fixed iteration under noisy conditions.

All iterative methods were initialized with the same estimate z(0), defined as a uniform image whose pixel values were set proportional to the average projection intensity, i.e.,

(18) $$z_{j}\left(0\right)={\left(\sum_{\ell =1}^{J}\sigma_{\ell }\right)}^{-1}\sum_{i=1}^{I}p_{i},\;j=1,2,\ldots ,J.$$

Using the two numerical phantoms, we first investigated the tunable parameters λ=(τ,ν) in the CDEM algorithm by constructing parameter planes based on the evaluation function in Equation (12) at the final iteration. These parameter planes enable visual identification of regions that yield the best reconstruction performance. We then examined the parameter adjustment method derived from the optimization principle and verified whether appropriate parameter selection improves reconstruction quality.

The parameter adjustment method derived from the optimization principle was evaluated using multiple noise realizations to confirm its robustness and effectiveness. For each of the K=8 projection datasets with the same noise level but different noise patterns, the projection data were generated as

(19) $$Y_{k}=Ae+\Delta_{k}\in R_{+}^{I},\;k=1,2,\ldots ,K,$$

where $\Delta =\left[\Delta_{1},\Delta_{2},\ldots ,\Delta_{K}\right]\in R^{I\times K}$ denotes a noise matrix whose columns $\Delta_{k}$ are independently generated noise vectors. Each $\Delta_{k}$ follows a normal distribution with variance chosen to achieve a specified SNR. The main experiments use 20 dB, while a supplementary sensitivity analysis is conducted at 10 dB. This modeling choice reflects the fact that noise in X-ray CT systems is well approximated by Gaussian statistics. The goal here is not to tune parameters to a specific noise realization, but rather to identify parameter values that remain effective across projection datasets sharing the same uncertainty level. The number of optimization iterations was fixed at N=30.

In the physical experiments, projection data were acquired using an X-ray CT scanner (Canon Medical Systems, Tochigi, Japan) and a phantom simulating the human body [20]. To investigate the noise suppression effect of the CDEM reconstruction method, which is particularly important in clinical applications requiring higher radiation doses, an abdominal phantom was used. The scanner was operated with a tube voltage of 80 kVp, tube current of 10 mA, and a scan time of 0.75 s per rotation. This setup corresponds to imaging conditions with a relatively high level of measurement noise compared with general abdominal protocols. Projections were obtained from 900 directions covering 360 degrees with 957 bins, and the data were subsampled to achieve 90 projection directions (*I* = 86,130) to simulate a more severe acquisition condition. Optimization was performed using projection data from four consecutive slices (K=4) and an evaluation function with N=60 iterations. The reconstructed image size was 674×674 (*J* = 454,276).

**Figure 2:**
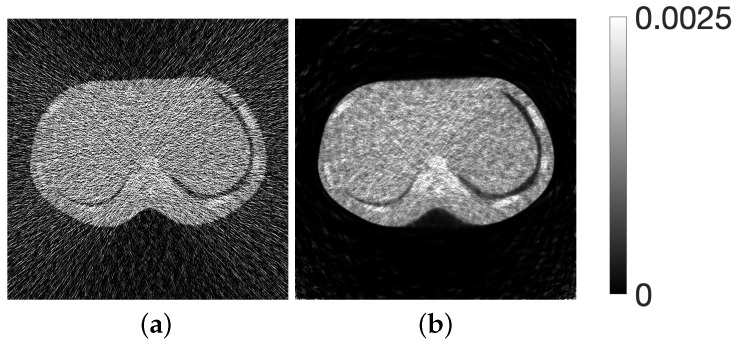
Images reconstructed from X-ray CT scanner projections using (**a**) FBP procedure and (**b**) MLEM method.

Figure 2 shows images created from one of the projection data slices using the filtered back-projection (FBP) method with a Shepp–Logan filter and the iterative reconstruction method using MLEM.

For parameter estimation, we employed the constrained nonlinear optimization solver fmincon provided in MATLAB R2024a (MathWorks, Natick, MA, USA).

The optimization problem defined in Equation (11) was solved using the interior-point algorithm implemented in fmincon. Unless otherwise noted, the initial parameter vector was set to λ(0)=(τ(0),ν(0))=(0.5,0.5), and simple bound constraints τ∈[−1.0,2.0] and ν∈[0.01,2.0] were imposed to ensure stability. Gradients were approximated using central finite differences with a stepsize of $10^{-4}$. The optimizer tolerances were set to OptimalityTolerance $=10^{-4}$, StepTolerance $=10^{-4}$, and FunctionTolerance $=10^{-4}$, with $\mathrm{MaxFunctionEvaluations}=10^{5}$ and MaxIterations=50. An output function was defined to record the iterative history of parameter updates ${\left\{\lambda^{\left(n\right)}\right\}}_{n}$ and the corresponding objective values at each iteration for post hoc analysis. The objective function $\frac{1}{K}\sum_{k=1}^{K}d\left(\cdot \right)$ was evaluated in parallel for k=1,2,…,K using the parfor construct in MATLAB’s Parallel Computing Toolbox to accelerate computation. Throughout the optimization, the internal state variables were monitored to detect any infeasible or divergent behavior (e.g., NaN or Inf values), ensuring numerical stability of the estimation process.

### 4.2. Experimental Results and Discussion

The following results correspond to the main experiments, conducted under the default conditions of SNR 20 dB and 90 projection directions.

**Figure 3:**
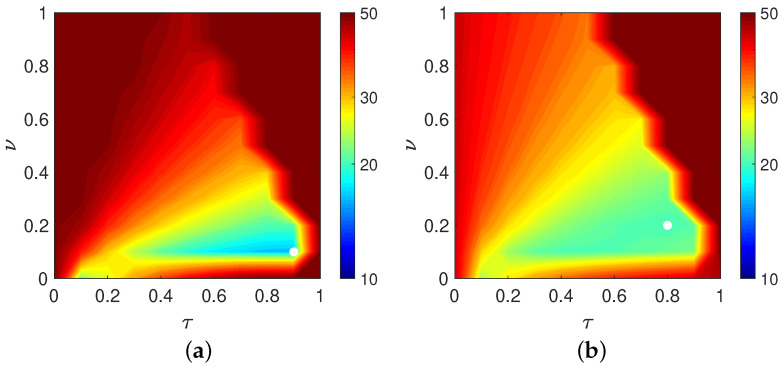
Parameter planes for the CDEM under the evaluation function in Equation (12), showing the behavior of the tunable parameters τ and ν. Panels (**a**) and (**b**) correspond to the Disc and the modified Shepp–Logan phantom, respectively. The white dot in each parameter plane indicates the parameter pair (τ,ν) that attains the minimum value of the evaluation function.

First, we examine how the choice of parameters affects reconstruction accuracy when the distance function is chosen as the Euclidean distance between the reconstructed and true images in Equation (12). Figure 3 shows the parameter planes for the Disc and the modified Shepp–Logan phantom, where the values of the evaluation function at the final iteration (60 iterations) are plotted over the parameter set λ. The parameter planes are constructed by sampling λ=(τ,ν) on a uniform grid with spacing 0.1, with τ on the horizontal axis and ν on the vertical axis. For visual clarity, higher values of the evaluation function are shown in red and lower values in blue. The parameter pair that attains the minimum value of the evaluation function is indicated by a white dot; the corresponding minimizers are (τ,ν)=(0.9,0.1) for the Disc phantom and (τ,ν)=(0.8,0.2) for the modified Shepp–Logan phantom. As can be seen in Figure 3, both the overall structure of the parameter plane and the location of the optimal parameter set depend strongly on the target phantom. The value of the evaluation function changes significantly across different parameter combinations, and certain regions exhibit unstable behavior. These results indicate that the optimal balance between the image-update dynamics and the auxiliary-variable dynamics strongly depends on the structural characteristics of the target object, highlighting the importance of appropriate parameter selection for robust and accurate reconstruction. Furthermore, Figure 3 visualizes the sensitivity of the evaluation function with respect to (τ,ν). The presence of sharply varying regions indicates that small parameter perturbations can lead to significant degradation in the evaluation value. This observation motivates the use of an optimization-based strategy to estimate robust and high-performing parameter pairs, rather than relying on exhaustive grid-based inspection.

**Figure 4:**
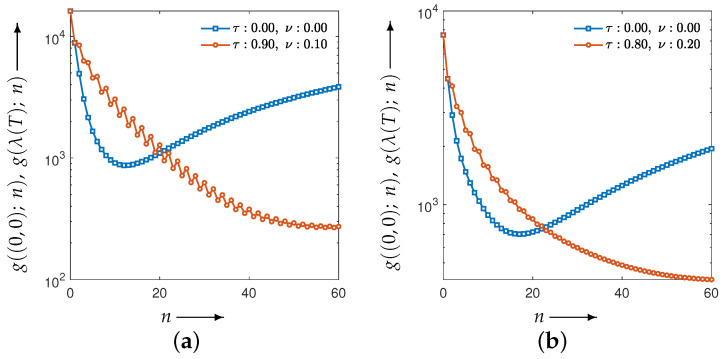
Plots of the evaluation function and the iteration process using the best parameter set (τ,ν) obtained from the parameter planes. The blue curve corresponding to (τ,ν)=(0,0) represents the MLEM method. For the best parameters, (**a**) the Disc yields (τ,ν)=(0.90,0.10), while (**b**) the modified Shepp–Logan phantom yields (τ,ν)=(0.80,0.20).

**Figure 5:**
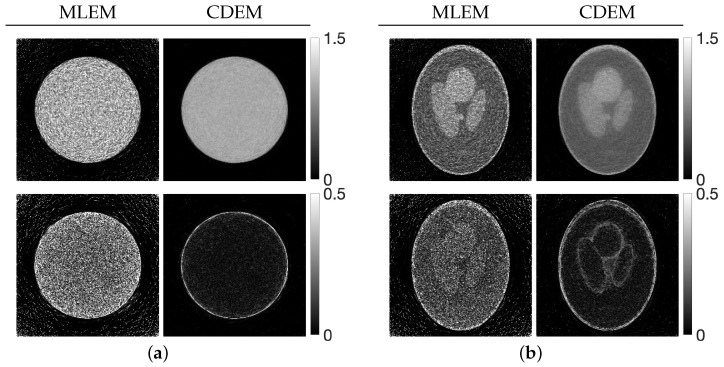
Images reconstructed obtained by CDEM using the best parameter set. Panels (**a**) and (**b**) correspond to the Disc and the modified Shepp–Logan phantom, respectively. The top row shows the reconstructed images, while the bottom row presents the difference images relative to the ground truth. For better visual inspection, the intensity range of the difference images has been adjusted.

Figure 4 plots the values of the evaluation function in Equation (12) at each iteration when the optimal parameter sets obtained from the parameter planes are applied. Figure 5 shows the reconstructed images (top row) at the final (60th) iteration for all methods, including MLEM, together with their difference images (bottom row) relative to the ground truth.

Although the CDEM reconstructions exhibit visually reduced noise effects at later iterations, Figure 4 provides insight into the temporal evolution of the evaluation function and reveals that both phantoms show noticeable oscillatory behavior in the early iterations. Moreover, when compared with MLEM, the early iteration evaluation values for CDEM are clearly higher, indicating that the parameter sets selected solely from the parameter planes do not fully extract the potential performance of CDEM. This suggests that relying on parameter planes alone is insufficient, as the optimal parameters for the final iteration do not necessarily ensure well-behaved and effective performance throughout the iterative process. In this sense, Figure 4 complements the parameter-plane analysis by demonstrating that appropriate parameter selection must account not only for the final evaluation value but also for stability and convergence behavior during the entire iteration process.

**Figure 6:**
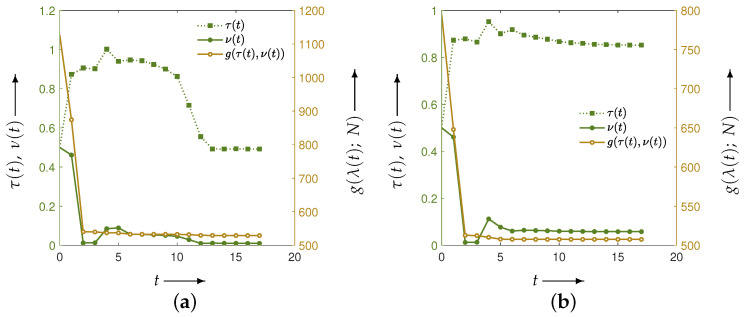
Parameters τ(t) and ν(t) (green iterative points, left axis) and the evaluation function g(λ(t);N) (brown iterative points, right axis) at each iteration *t* in the optimization process of the evaluation function for phantoms (**a**) Disc and (**b**) modified Shepp–Logan. The distance function is the Euclidean distance between the reconstructed image and the true image.

Next, for the two phantoms, Figure 6 illustrates the evolution of the parameters and the evaluation function *g* obtained by solving the optimization problem associated with the set in Equation (11). The horizontal axis represents the iteration index t=0,1,2,… of the optimization algorithm. The green points denote the parameter values λ(t), referenced against the left vertical axis, while the brown points represent the corresponding evaluation values g(λ(t);N), referenced against the right vertical axis. The algorithm terminates either when the maximum number of iterations is reached or when the change in the parameter vector falls below a prescribed tolerance; the iteration at which termination occurs is denoted by *T*.

Independent of the phantom type, the parameter vector λ(t) changes over the course of the iteration process, and the evaluation function g(λ(t);N) decreases monotonically. However, the final parameters λ(T) differ across phantoms, yielding (τ,ν)=(0.49,0.01) for the Disc and (τ,ν)=(0.85,0.06) for the modified Shepp–Logan phantom.

**Figure 7:**
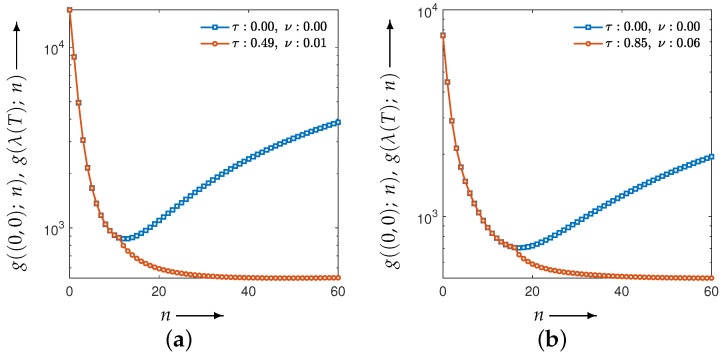
Evaluation function values and iteration trajectories after parameter adjustment by optimization. The blue curve corresponds to the MLEM method. Panels (**a**) and (**b**) show the results for the Disc and the modified Shepp–Logan phantom, respectively.

Figure 7 plots the evaluation function values versus the iteration index *n*. The blue points correspond to the MLEM method, which is the special case of CDEM with the fixed parameter λ=(0,0). This curve is shown as a reference and is independent of the parameter-optimization process. The agreement of the initial evaluation values between MLEM and CDEM is due to the use of identical initial image values and the initialization of the auxiliary projection variable with the measured projection *p*. Moreover, the value g(λ(T);N) at N=30 corresponds to the value at the final optimization iteration t=T in Figure 6, and it coincides with the CDEM evaluation value at iteration n=30 in Figure 7.

**Figure 8:**
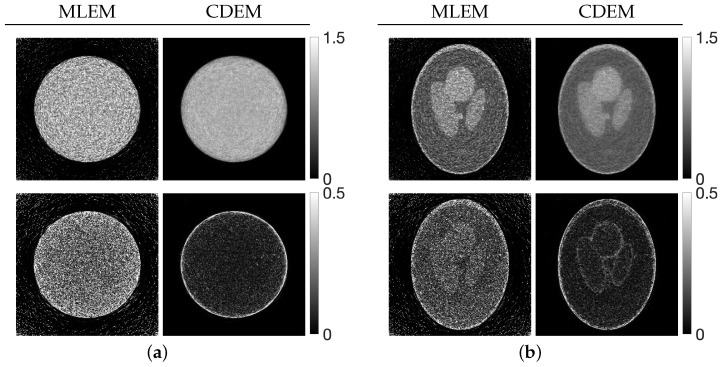
Images reconstructed using MLEM and CDEM (**top**) and corresponding difference images (**bottom**) defined by the distance function for phantoms (**a**) Disc and (**b**) modified Shepp–Logan. The distance function is the Euclidean distance between the reconstructed image and the true image.

**Figure 9:**
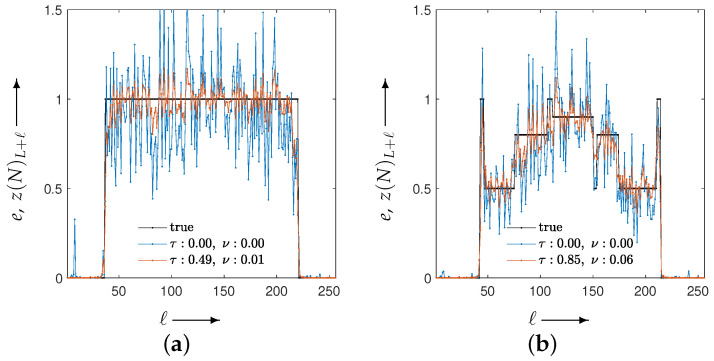
The density profile along the column direction (ℓ=1,2,…,256), fixed at the 102nd row (L=101×256), for images reconstructed using MLEM and CDEM for phantoms (**a**) Disc and (**b**) modified Shepp–Logan. The black, blue, and red lines represent the true values, MLEM, and CDEM, respectively. The distance function is the Euclidean distance between the reconstructed image and the true image.

Figure 8 shows, at iteration *N*, the reconstructed images produced by MLEM and by CDEM with the optimized parameters λ(T), together with the corresponding difference images relative to the true phantom. Compared with MLEM, the CDEM reconstructions exhibit reduced noise and improved structural preservation. The density profiles along the column direction of the images are plotted in Figure 9.

Next, we determine optimal parameter values under several choices of evaluation function and identify which criterion is most suitable for achieving effective noise suppression in tomographic reconstruction. Although the image domain discrepancy in Equation (12) is advantageous when ground-truth images are available, such information is rarely accessible in practical settings. We therefore turn our attention to projection-based criteria, namely the Euclidean distance and KL-divergence in Equations (13) and (14), as well as the coupled-system–oriented measures in Equations (16) and (17). For each of these distance functions, we first perform parameter optimization to obtain the corresponding best-performing parameter set and then compare the resulting reconstructions in order to identify the most effective evaluation function for noise-suppressed tomographic image reconstruction.

**Figure 10:**
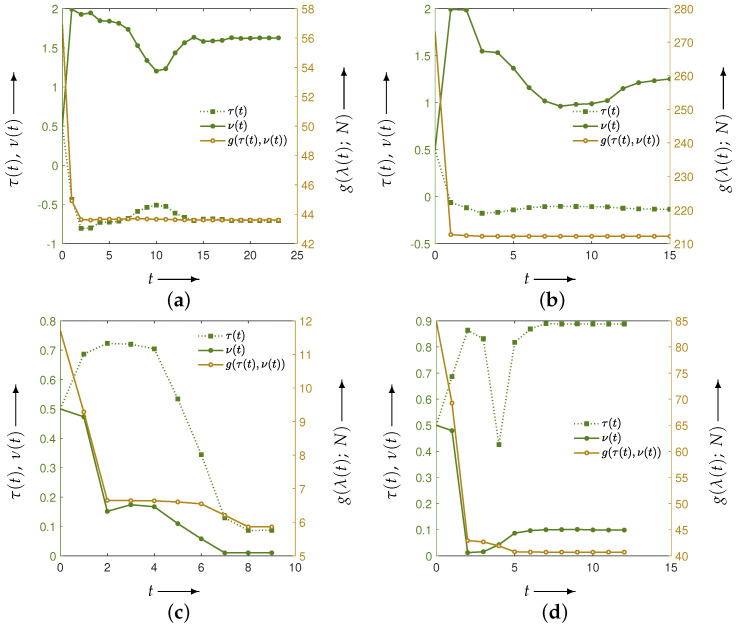
The parameters τ(t) and ν(t) (green iterative points, left axis) and the evaluation function g(λ(t);N) (brown iterative points, right axis) during each iteration *t* in the optimization process for a modified Shepp–Logan phantom. The distance function is the (**a**) Y2N, (**b**) YKL, (**c**) C2N, and (**d**) CKL between forward and measured projections.

**Figure 11:**
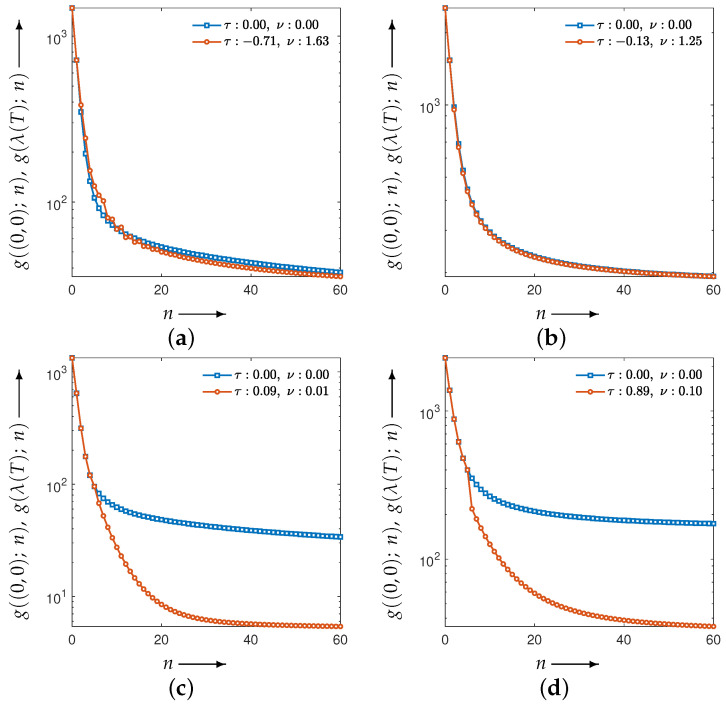
Change in the evaluation functions g((0,0);n) and g(λ(T);n) with the number of iterations *n* (shown as blue and red iterative points, respectively) for a modified Shepp–Logan phantom. The distance functions are the (**a**) Y2N, (**b**) YKL, (**c**) C2N, and (**d**) CKL between forward and measured projections.

**Figure 12:**
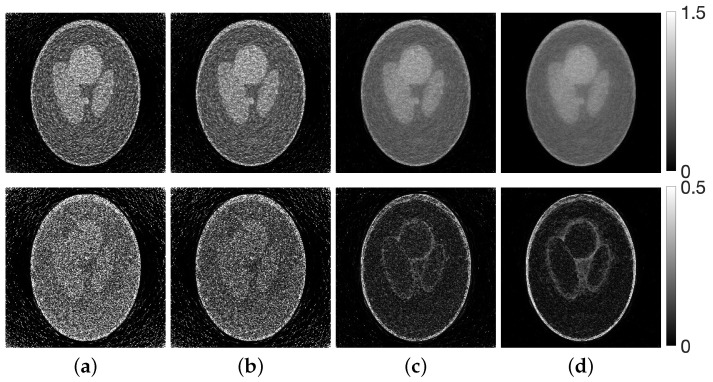
Images reconstructed using CDEM (with parameters λ(T)) (**top**) and the corresponding difference images (**bottom**) for a modified Shepp–Logan phantom. The distance function between forward and measured projections are (**a**) Y2N, (**b**) YKL, (**c**) C2N, and (**d**) CKL.

Figure 10 shows the evolution of the parameters and the evaluation function *g* obtained by solving the optimization problem associated with the set in Equation (11). Figure 11 illustrates the iterative behavior of each evaluation function when the initial parameters λ(0) and the optimized parameters λ(T) are applied to the CDEM, and Figure 12 presents the reconstructed images at iteration *N*. Since the modified Shepp–Logan phantom is widely used as a benchmark for tomographic reconstruction, these figures show the results for the modified Shepp–Logan phantom as representative examples.

Table 1 and Table 2 summarize, for each distance function, the optimized parameter values λ(T), the quantitative image-quality metrics peak signal to noise ratio (PSNR) and multiscale structural similarity index measure (MSSIM), and the standard deviation between the reconstructed and true images when the optimization process reaches iteration *T*. In particular, Table 1 demonstrates how the choice of evaluation function directly influences the resulting parameter values and reconstruction performance for the Disc phantom. The projection-based criteria Y2N and YKL do not yield improvements over MLEM in terms of PSNR, MSSIM, or the standard deviation. In contrast, C2N and CKL explicitly balance the consistency with the auxiliary projection and the fidelity to the measured projection, resulting in a marked increase in PSNR and MSSIM together with a substantial reduction in the standard deviation. These results indicate that evaluation functions consistent with the coupled dynamical structure are important for extracting the noise-suppression capability of CDEM, even for a simple homogeneous object such as the Disc.

As can be seen in Figure 10, the parameter trajectories remain smooth and bounded for all four evaluation functions, and the corresponding values of the evaluation function decrease consistently throughout the optimization process. However, the evaluation plots in Figure 11 show that the projection-based criteria in Equations (13) and (14) fail to achieve any performance improvement over MLEM. As confirmed in Figure 12, the reconstructed images obtained using Equations (13) and (14) exhibit insufficient noise suppression. Moreover, the optimal parameters selected by these two criteria do not lead to performance improvements over MLEM in terms of PSNR, MSSIM, or the standard deviation. This tendency is observed not only for the modified Shepp–Logan phantom but also for the Disc, as shown in Table 1 and Table 2.

In contrast, the composite criteria C2N and CKL substantially outperform MLEM, as clearly demonstrated in Figure 11. The reconstructed images in Figure 12 also show that these criteria achieve visibly superior noise reduction and structural fidelity. The quantitative results in Table 1 and Table 2 further confirm this trend: for both the Disc and the modified Shepp–Logan phantom, C2N and CKL yield the highest PSNR, MSSIM, and lowest standard deviation. Notably, within the experimental settings considered in this study, the CKL criterion demonstrated performance comparable to that of the ground-truth-based measure (Equation (12)). These results suggest that evaluation functions consistent with the coupled system dynamics can provide effective parameter selection without requiring ground truth, although the comparative performance may depend on noise level, geometry, and iteration number.

**Figure 13:**
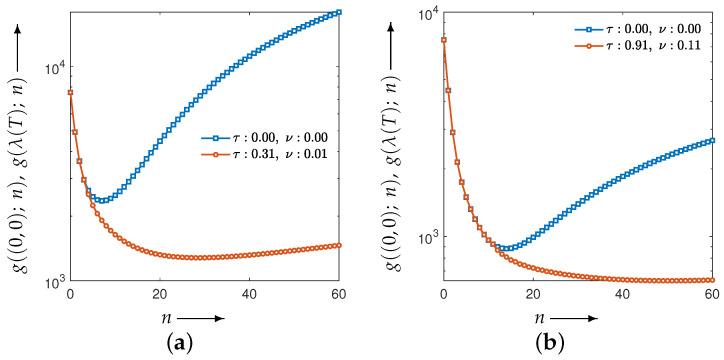
Evaluation function values and iteration trajectories after parameter adjustment by optimization for the modified Shepp–Logan phantom. The blue curve corresponds to the MLEM method. (**a**) shows the result at SNR of 10 dB, whereas (**b**) shows the result with 45 projections.

To further verify the effectiveness of the proposed method under more challenging conditions, we conducted supplementary experiments using the modified Shepp–Logan phantom. In these supplementary experiments, we considered adverse conditions by either increasing the noise level to an SNR of 10 dB or reducing the number of projections to 45, while keeping the other factor at its default value. The change in the evaluation function with respect to the iteration number, where the distance function is the Euclidean distance between the reconstructed image and the true image, is shown in Figure 13.

**Figure 14:**
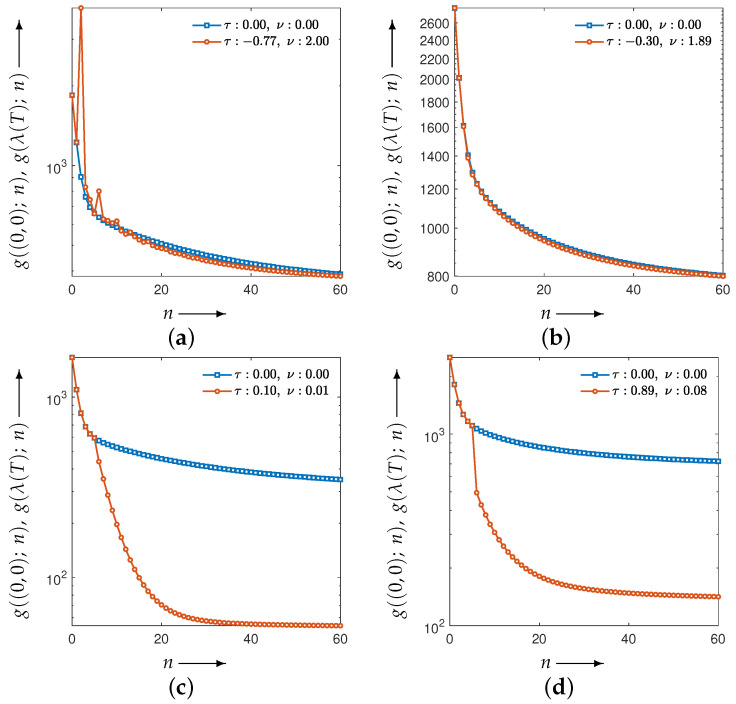
Change in the evaluation functions g((0,0);n) and g(λ(T);n) with the number of iterations *n* (shown as blue and red iterative points, respectively) for a modified Shepp–Logan phantom at SNR of 10 dB. The distance functions are the (**a**) Y2N, (**b**) YKL, (**c**) C2N, and (**d**) CKL between forward and measured projections.

**Figure 15:**
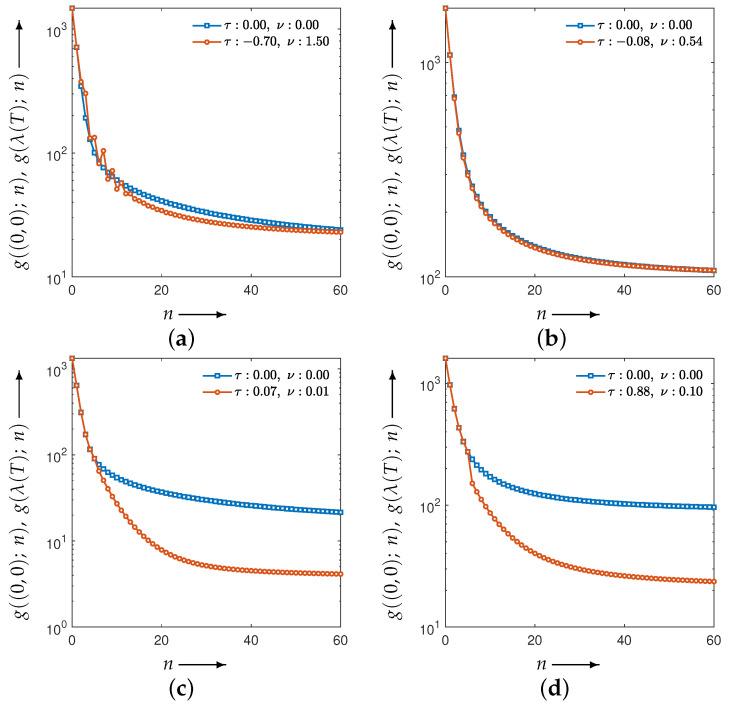
Change in the evaluation functions g((0,0);n) and g(λ(T);n) with the number of iterations *n* (shown as blue and red iterative points, respectively) for a modified Shepp–Logan phantom with 45 projections. The distance functions are the (**a**) Y2N, (**b**) YKL, (**c**) C2N, and (**d**) CKL between forward and measured projections.

Subsequently, under adverse conditions of either an SNR of 10 dB or 45 projections, we determined the optimal parameter values for several choices of evaluation function and identified which criteria are most suitable for achieving effective noise suppression in tomographic reconstruction. The corresponding results are shown in Figure 14 and Figure 15, respectively.

Consistent with the previous results, these experiments indicate that effective parameter estimation yields good performance even under these settings. Moreover, with respect to the choice of evaluation function, C2N and CKL continue to preserve and adequately extract the performance of the proposed method, serving as suitable criteria for achieving effective noise suppression.

**Figure 16:**
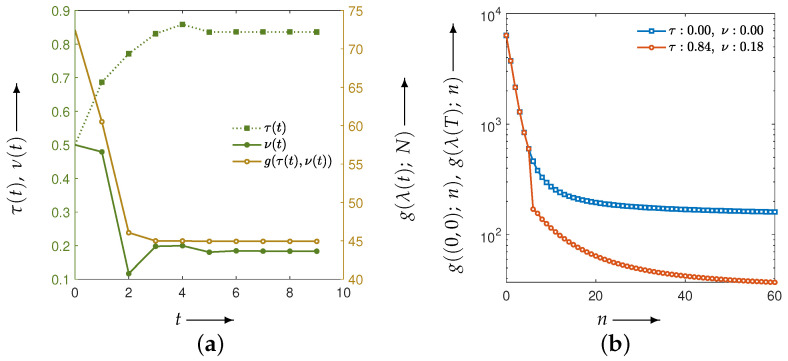
(**a**) The parameters τ(t) and ν(t) (green iterative points, left axis) and the evaluation function g(λ(t);N) (brown iterative points, right axis) at each iteration *t* during the optimization process of the evaluation function using projections from an X-ray CT scanner, and (**b**) the change in the evaluation function g((0,0);n) and g(λ(T);n) (shown as blue and red iterative points, respectively) over iteration count *n*. The distance function is the CKL between forward and measured projections.

**Figure 17:**
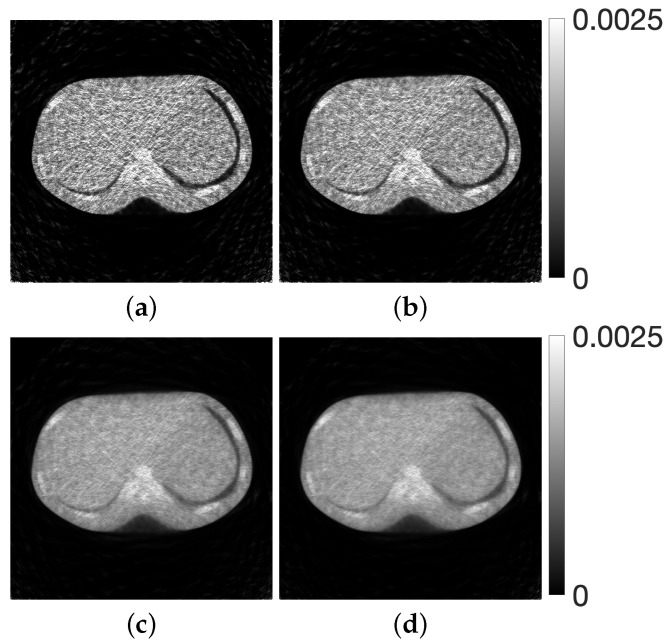
Images reconstructed using CDEM (parameters λ(T)) from projections obtained with an X-ray CT scanner. The distance functions between forward and measured projections are (**a**) Y2N, (**b**) YKL, (**c**) C2N, and (**d**) CKL.

**Figure 18:**
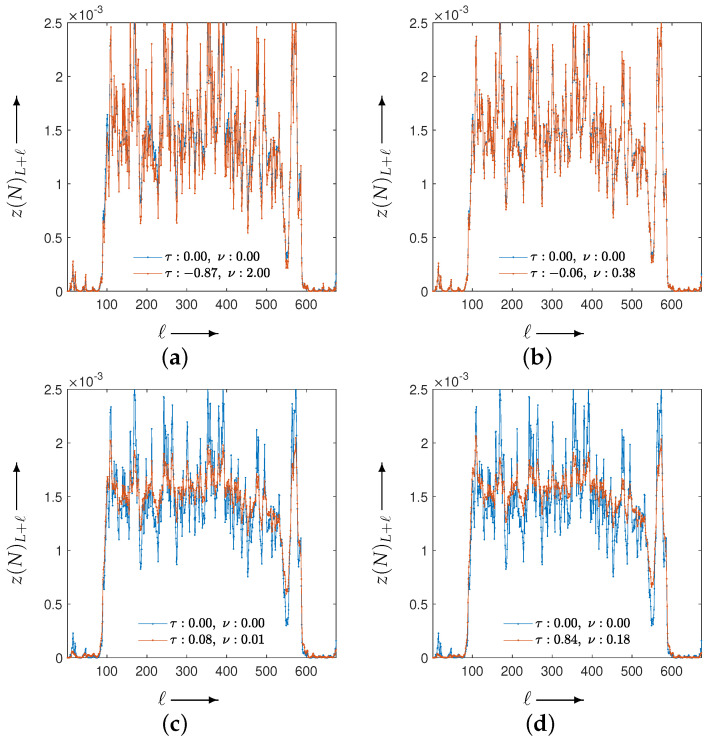
Density profile along the column direction (ℓ=1,2,…,674), fixed at the 224th row (L=223×674), in images reconstructed using MLEM and CDEM from projections obtained with an X-ray CT scanner. The blue and red lines represent MLEM and CDEM, respectively. The distance functions between forward and measured projections are (**a**) Y2N, (**b**) YKL, (**c**) C2N, and (**d**) CKL.

Finally, on the basis of the numerical experiments where the composite criteria C2N and CKL were identified as the most effective evaluation functions for parameter optimization in CDEM, we conducted experiments using a physical phantom. Unlike the numerical phantoms, the true image is not available in this setting, and therefore the reconstruction quality must be assessed solely from measured projection data. Figure 16 shows the optimization process and the transition of the evaluation function when the proposed method was applied to the projection data obtained from the X-ray CT scanner, using CKL as the distance function between the forward and measured projections. The reconstructed images and the corresponding density profiles, including those obtained with other distance functions, are presented in Figure 17 and Figure 18, respectively.

As shown in Figure 16, the results for the CKL criterion, which demonstrated the best performance in the numerical phantom experiments, indicate that the evaluation function decreases monotonically throughout the optimization process and consistently outperforms MLEM. Furthermore, Figure 17 and Figure 18 show that the composite criteria C2N and CKL yield substantially better reconstruction quality than the projection-based criteria Y2N and YKL. These findings demonstrate that, with appropriate parameter selection and a suitable evaluation function, the CDEM fully exploits its coupled dynamical structure for both numerical and physical phantoms, resulting in markedly improved noise-suppression performance compared with conventional MLEM. This suggests that, in practical clinical applications, the proposed approach has the potential to enable significant reductions in radiation dose while maintaining reliable image quality.

## 5. Conclusions

We investigated an optimization-based parameter selection approach for the coupled dynamical systems previously proposed as iterative reconstruction methods with tunable parameters. Focusing on the CDEM formulation, two adjustable parameters appearing in the update rules were optimized through numerical experiments. The results demonstrated that parameter optimization based on the evaluation function, particularly when using the coupled distance measures C2N and CKL defined in the proposed algorithm, yields the most effective reconstruction performance. One of the key findings is that, although the proposed algorithm uses distance functions defined solely between the forward and measured projections, its performance is comparable to that achieved using ground-truth-based image domain distances. This indicates that the proposed criteria successfully capture reconstruction quality without requiring access to the true image. Physical phantom experiments further confirmed that the proposed parameter selection approach achieves results consistent with those obtained in numerical simulations, thereby demonstrating that the method effectively improves image quality in iterative reconstruction using CDEM and is applicable to practical CT imaging scenarios. This approach can be extended to other coupled dynamical formulations and inverse problems. In the present study, however, we focused on the EM-type algorithm. In contrast, the robust parameter range and a practical parameter tuning strategy for the MA-type algorithm have not yet been rigorously evaluated. In addition, the performance of the proposed method depends on the choice of parameters and on the imaging conditions, which may limit its robustness without appropriate tuning. Furthermore, the development of faster automatic parameter selection; validation under varying noise levels and view numbers; applications to low-dose CT imaging; and additional clinical applications such as head and chest imaging beyond abdominal CT and extension to other tomographic modalities, including positron emission and single-photon emission computed tomography, remain important subjects for future work.

## Figures and Tables

**Table 1:** Performance evaluation of images reconstructed using MLEM and CDEM (with parameters λ(T)) for the Disc. The distance functions are (a) Y2N, (b) YKL, (c) C2N, and (d) CKL between forward and measured projections, and (e) Euclidean distance between the reconstructed and true images.

Method	Dist. Func.	τ	ν	PSNR	MSSIM	Std. Dev.
MLEM	—	0	0	12.3	0.549	0.243
	(a)	−0.64	1.59	9.56	0.480	0.332
	(b)	−0.12	1.51	11.8	0.536	0.256
CDEM	(c)	0.09	0.01	19.8	0.810	0.102
	(d)	0.89	0.09	21.9	0.886	0.080
	(e)	0.49	0.01	21.0	0.866	0.089

**Table 2:** Performance evaluation of images reconstructed using MLEM and CDEM (with parameters λ(T)) for modified Shepp–Logan phantom. The distance functions are (a) Y2N, (b) YKL, (c) C2N, and (d) CKL between forward and measured projections, and (e) Euclidean distance between the reconstructed and true images.

Method	Dist. Func.	τ	ν	PSNR	MSSIM	Std. Dev.
MLEM	—	0	0	15.2	0.696	0.173
	(a)	−0.71	1.63	12.3	0.622	0.241
	(b)	−0.13	1.25	14.6	0.680	0.185
CDEM	(c)	0.09	0.01	21.0	0.874	0.089
	(d)	0.89	0.10	21.3	0.899	0.086
	(e)	0.85	0.06	21.1	0.890	0.087

## Data Availability

The original contributions presented in this study are included in the article material. Further inquiries can be directed to the corresponding author.
